# Alterations in Proteostasis System Components in Peripheral Blood Mononuclear Cells in Parkinson Disease: Focusing on the HSP70 and p62 Levels

**DOI:** 10.3390/biom12040493

**Published:** 2022-03-24

**Authors:** Julia D. Vavilova, Anna A. Boyko, Natalya I. Troyanova, Natalya V. Ponomareva, Vitaly F. Fokin, Ekaterina Y. Fedotova, Maria A. Streltsova, Sofya A. Kust, Maria V. Grechikhina, Olga A. Shustova, Tatyana L. Azhikina, Elena I. Kovalenko, Alexander M. Sapozhnikov

**Affiliations:** 1 Shemyakin and Ovchinnikov Institute of Bioorganic Chemistry, Russian Academy of Sciences, 117997 Moscow, Russia; boyko_anna@mail.ru (A.A.B.); troyanatali@gmail.com (N.I.T.); mstreltsova@mail.ru (M.A.S.); sonya.erokhina@gmail.com (S.A.K.); marygrec@mail.ru (M.V.G.); olga_shustova@list.ru (O.A.S.); tatazhik@ibch.ru (T.L.A.); lenkovalen@mail.ru (E.I.K.); amsap@mail.ru (A.M.S.); 2 Research Center of Neurology, 125367 Moscow, Russia; ponomare@yandex.ru (N.V.P.); fvf@mail.ru (V.F.F.); ekfedotova@gmail.com (E.Y.F.)

**Keywords:** Parkinson disease, PBMC, proteostasis, HSP70, autophagy, p62

## Abstract

Parkinson disease (PD) is attributed to a proteostasis disorder mediated by α-synuclein accumulating in a specific brain region. PD manifestation is often related to extraneuronal alterations, some of which could be used as diagnostic or prognostic PD biomarkers. In this work, we studied the shifts in the expression of proteostasis-associated chaperones of the HSP70 family and autophagy-dependent p62 protein values in the peripheral blood mononuclear cells (PBMC) of mild to moderate PD patients. Although we did not detect any changes in the intracellular HSP70 protein pool in PD patients compared to non-PD controls, an increase in the transcriptional activity of the stress-associated *HSPA1A/B* and *HSPA6* genes was observed in these cells. Basal p62 content was found to be increased in PD patients’ PBMC, similarly to the p62 level in substantia nigra neural cells in PD. Moreover, the spontaneous apoptosis level was increased among PBMC and positively correlated with the p62 intracellular level in the PD group. A combined *HSPA6*- and p62-based analysis among 26 PD patients and 36 age-matched non-PD controls pointed out the diagnostic significance of these markers, with intermediate sensitivity and high specificity of this combination when observing patients diagnosed with PD.

## 1. Introduction

As a multifactor neurodegenerative disease, Parkinson disease (PD) is mainly characterized by progressive motor impairment, leading to a disruption of the normal lifestyle of more than 1% of people over the age of 60 [1]. PD is attributed to a selective loss of dopaminergic neurons in the substantia nigra as a result of α-synuclein accumulation and Lewy body formation, which leads to the disruption in the substantia nigra pars compacta and, to a lesser extent, in other regions of the brain [2]. Motor dysfunctions in PD include tremor at rest, bradykinesia, stiffness, postural instability, and a wide range of non-motor symptoms (mental, cognitive, etc.) are often accompanying PD [3]. It is known that α-synuclein aggregates accumulate in the neurons due to the imbalanced cellular proteostasis in PD [4]. The manner of α-synuclein degradation in neurons remains controversial.

Proteostasis pathways, basically chaperone-mediated autophagy (CMA) and macroautophagy, have been suggested to contribute to the α-synuclein turnover [5]. CMA dysregulation, related to an altered expression of autophagic genes, is observed not only in familial but also in the sporadic forms of PD [6]. Heat shock proteins are induced in various pathological conditions in the brain, including stroke, neurodegenerative disease, epilepsy, and trauma [7]. Heat shock proteins of the 70 kDa family (HSP70) ensure folding, refolding, degradation, and elimination of damaged and aggregated substrates, including through the mechanism of CMA by constitutively expressed Hsc70 protein [8,9]. Hsc70 recognizes proteins that exhibit a KFERQ-like motif and directs them into lysosomes for degradation [10]. Post-mortem brain samples from PD patients reveal a decreased level of Hsc70 in the substantia nigra pars compacta and amygdala compared to healthy controls [11]. In addition to Hsc70 protein, which is predominant in the cytosol, the HSP70 pool contains other proteins, such as the cytosolic stress-induced Hsp70-1, the organelle-specific mitochondrial HSP75/mortalin, and ER/HSP78/BIP proteins. All these proteins are encoded by different *HSPA* genes and play a role in the protein homeostasis maintenance, in supporting organelles’ functioning, and in the regulation of inflammatory signaling pathways [12]. Hsp70-1 has two protein isoforms, Hsp70-1a and Hsp70-1b encoded by *HSPA1A* and *HSPA1B* genes, respectively. These isoforms are thought to be interchangeable by their functions because of a similarity in their gene structures [13]. Basal expression of *HSPA1A* and *HSPA1B* genes differs in most tissues; however, similar basal levels of *HSPA1A* mRNA were observed in the blood and the brain [14]. HSP70 overexpression using viral vectors improved survival of neurons and astrocytes in stroke models and, thus, it was evidence of a neuroprotective role of HSP70 [15]. Another stress-induced HSP70 protein, Hsp70B’, is encoded by the *HSPA6* gene. The *HSPA6* gene is found exclusively in the human genome and, therefore, has not been studied in animal models of neurodegenerative diseases [16]. *HSPA6* is not detected at all in intact cells and its expression is far more strictly related to stress compared to the other *HSPA* genes, especially in hyperthermia [17]. The involvement of Hsp70B’ in PD remains vague. It was shown that the experimental blockage of the CMA pathway upregulated macroautophagy [18]. Macroautophagy is essential for the removal of long-lived proteins and dysfunctional organelles in eukaryotic cells to prevent possible toxicity and cell death. The basic component of macroautophagy process is p62 protein, also called sequestosome 1 (SQSTM1). It selectively links ubiquitinated proteins to the autophagic machinery, which enables their degradation in the lysosome [19]. The p62 protein, together with polyubiquitinated proteins, incorporates into the mature autophagosome to be degraded in autolysosomes. The p62 has been detected in ubiquitinated protein aggregates, including Lewy bodies in PD [20]. Since the accumulation of this protein is intensified in cells when autophagy is inhibited and declines when autophagy is induced, the p62 level may be considered as an indicator of autophagic degradation [21].

Clinical symptoms of PD generally manifest when the amount of defective dopaminergic neurons reaches 60–80% [22]; thus, the early diagnosis of PD remains important. Certain shifts of the peripheral blood indicators (biochemical, phenotypic, immunological) in association with PD manifestation have been already described, which could make the extraneuronal cell characteristics worth considering as diagnostic and/or prognostic markers of the disease [23,24]. The PD patient’s immune status is characterized by reduced immunosenescence compared with age-matched healthy donors and is similar to young individuals [25,26]. Impaired dopamine synthesis, abnormal α-synuclein metabolism, and mitochondrial dysfunctions, i.e., those changes that are involved in the pathogenesis of PD, are registered in peripheral T cells of PD patients [24,27,28]. Autophagy dysfunction caused by autophagy-related protein alterations, mainly autophagic-lysosomal proteins, was reported in PBMC from PD patients [24,29,30]. Significant differences in gene expression in peripheral blood cells can be detected even at early stages of PD compared to healthy donors [31], which allows us to consider PBMC as an available biomaterial for identifying possible disease biomarkers.

In this study, we performed a comparative analysis of proteostasis system components, focusing on HSP70 proteins, basal levels of the transcriptional activity of the encoding *HSPA* genes, and intracellular macroautophagy-associated p62 protein value in PBMC of patients with idiopathic mild to moderate PD and age-matched healthy donors.

## 2. Materials and Methods

### 2.1. Participants and Ethics’ Statement

The study was approved by the RCN Local Medical Ethics Committee (No. 11/14 19 November 2014). The written, informed consent was provided by all participants. A total of 26 patients with idiopathic Parkinson disease (PD patients) and 36 age-matched non-PD controls (healthy donors (HD)) participated in the study. The demographic and clinical characteristics of donors are shown in Table 1. The group of PD patients and HDs did not differ in age or gender. PD was diagnosed according to the “Parkinson’s Disease Society Brain Bank” criteria [32]. A scale of Hoehn and Yahr was applied for determination of the stages of the disease; stages were ≤3. The severity of clinical symptoms was evaluated by the unified PD scale: MDS-UPDRS (the Movement Disorder Society-Unified Parkinson’s Disease Rating Scale). All patients were treated with carbidopa/levodopa in combination with dopamine agonist therapy, in the absence of immunosuppressive therapy. Acute infectious or autoimmune diseases identified within a month before blood sampling were criteria of exclusion from the PD patients’ cohort. HDs were examined to be free of psychiatric and neurological conditions. Exclusion criteria for HD were a history of neurological and psychiatric diseases (cerebrovascular diseases, hypertension, epilepsy, and endogenous disorders) and any kind of memory impairment.

### 2.2. Isolation of PBMC and PMN from Donor Peripheral Blood

Venous blood was collected in vacuum tubes with EDTA (APEXLAB, Moscow, Russian). The procedure of cell isolation was performed within 0.5 h after blood sampling. Cells’ fractions with separated polymorphonuclear and mononuclear leukocytes were obtained by density gradient centrifugation using PolymorphPrep medium (Axis-Shield, Oslo, Norway) following the manufacturer’s instructions. PMN and PBMC were washed twice in Dulbecco’s phosphate buffer saline (DPBS) and then resuspended at a concentration of 2 × 10^6^ cells/mL in assay medium (RPMI-1640 medium (PanEco, Moscow, Russia supplemented with 2 mM of l-glutamine, 15 mM of HEPES, and 2% fetal calf serum (FCS, HyClone Labs, Logan, UT, USA).

### 2.3. HSP70 Immunolabeling

Intracellular HSP70 levels were determined by indirect immunofluorescent staining. The procedure of heating (43 °C for 10 min) in a constant-temperature water bath was performed for a number of individual samples. Before intracellular labeling, the cells were fixed and permeabilized with Cytofix/Cytoperm solution (BD Biosciences, San Jose, CA, USA). After two washes with DPBS containing 0.2% BSA and 0.1% Triton X-100, fixed-permeabilized cells were stained with the primary monoclonal antibodies, BRM22 (Sigma-Aldrich, St. Louis, MO, USA), recognizing both stress-induced Hsp70-1 and constitutive Hsc70, or with Hsp70-1-specific antibody C92F3A-5 (Stressgen, Enzo Life Sciences, Farmingdale, NY, USA) and then labeled with PE-conjugated secondary IgG Fab-fragments. Intracellular HSP70 levels were determined by flow cytometry analysis as mean fluorescence intensity (MFI) corrected for background fluorescence of the control samples (cells treated with secondary PE-conjugated antibodies only) by the formula: (MFIsample/MFIcontrol) − 1.

### 2.4. RNA Extraction, cDNA Synthesis, and qRT-PCR

Total RNA was from 6 × 10^6^ cells of PMN or PBMC using AllPrep DNA/RNA Kits (QIAGEN, Germantown, MD, USA) according to the manufacturer’s instructions. The cDNA synthesis was performed using an oligo-dT primer and MINT Reverse Transcriptase (Evrogen, Moscow, Russia) following the manufacturer’s instructions. The design of PCR primers (5′–3′) for real-time PCR was performed using NCBI primer blast [33]. As the sequences of *HSPA1A* and *HSPA1B* are highly homologous, primers specific for *HSPA1A* and *HSPA1B* were chosen in the 3′-UTR (3′-untranslated regions) of *HSPA1A* and *HSPA1B* (Supplementary Figure S2). The ability of the primer sets *HSPA1A* for-rev and *HSPA1B* for-rev to discriminate between *HSPA1A* and *HSPA1B* was confirmed by Sanger sequencing of PCR fragments obtained by amplification of the cDNA template with the appropriate primers. The list of the primer pairs used in the study is given in Supplementary Table S1.

Real-time PCR was carried out using a LightCycler 480 real-time PCR detection system (Roche Diagnostics, Mannheim, Germany). The procedure was described in detail earlier [34].

### 2.5. Apoptosis Measurement

PBMC were resuspended in RPMI 1640 plus 10% fetal calf serum at a concentration of 0.5 × 10^6^ cells/mL per well. Cells were incubated at 37 °C, 5% CO_2_, for 20 h for the evaluation of spontaneous apoptosis. Apoptosis was detected by annexinV-AF647 (Invitrogen, San Jose, CA, USA) and propidium iodide (PI, Sigma-Aldrich, St. Louis, MO, USA). PBMC were analyzed by flow cytometry after incubation with annexinV-AF647 for 15 min at RT and the addition of PI solution (2 μg/mL). AnnexinV-positive, PI-negative cells were considered to be undergoing apoptosis.

### 2.6. Flow Cytometry

Flow cytometry analysis was carried out on a FACSCalibur flow cytometer (BD Biosciences, Franklin Lakes, NJ, USA). Data were analyzed using CellQuest ver. 3.4 (BD Biosciences, Franklin Lakes, NJ, USA) and FlowJo software version X (TreeStar Williamson Way, Ashland, OR, USA).

### 2.7. The p62 ELISA

The quantitative p62 measurement was carried out in PBMC samples that were lysed in manufacturer’s buffer (samples stored at −80 °C before analysis) using a highly validated, quantitative p62 ELISA kit (ADI-900-212-0001, Enzo Life Sciences, NY, USA,) according to the manufacturer’s instructions.

### 2.8. Statistical Analysis

Statistical analysis was performed using GraphPad Prism 7 software (StatSoft Inc., Tulsa, OK, USA). T-tests and Mann–Whitney U test were performed for normally and abnormally distributed data, respectively. Correlation analysis was carried out using Pearson’s correlation test for data with normal distribution and Spearman’s correlation test for abnormally distributed data. Receiver operating characteristic (ROC) curves were made, and the area under the curve (AUC) was calculated to evaluate the predictive sensitivity and specificity of PBMC *HSPA* gene expression and p62 intracellular level for PD diagnosis. The cutoff value for the ROC analysis was determined using the Youden Index. A K Nearest Neighbor (KNN) algorithm was applied for representation and prediction of two simultaneous, independent indicators’ significance in distinguishing PD and non-PD control. A *p*-value < 0.05 was considered statistically significant.

## 3. Results

### 3.1. The Intracellular HSP70 Pool in PBMC Does Not Differ between PD Patients and Healthy Donors

**Figure 1:**
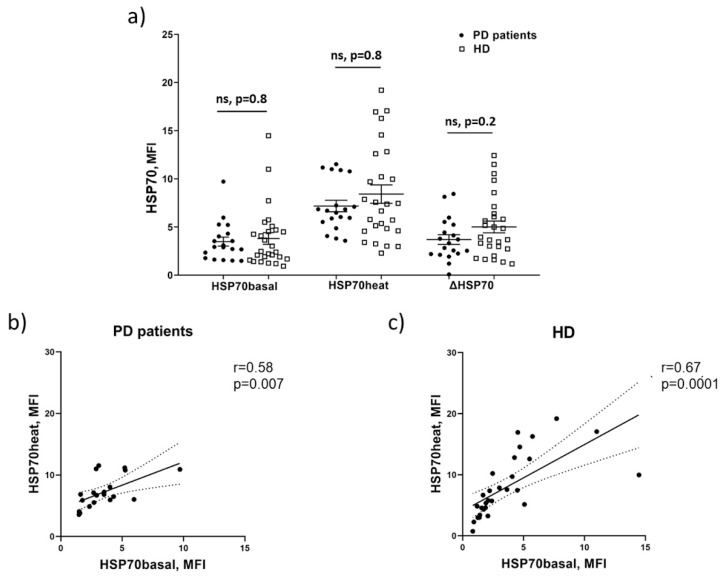
The level of the intracellular HSP70 pool (Hsc70 + Hsp70) in PBMC. (**a**) Basal (HSP70basal), heating-induced (HSP70heat) HSP70 levels, and ΔHSP70 in PD patients (*n* = 19) and HDs (*n* = 27); ΔHSP70 values were calculated as ΔHSP70 = HSP70heat − HSP70basal; (**b**) a correlation analysis between HSP70basal and HSP70heat levels in PBMC isolated from PD patients (**c**) from HDs. The results are presented as means of fluorescence intensity, corrected for background fluorescence of the control sample (MFI). Solid lines, mean ± SEM; ns, non-significant. (**b**,**c**) Pearson’s correlation coefficient was used; dotted line, 95% confidence interval.

The intracellular HSP70 pool was measured in intact PBMC (HSP70basal) of PD patients and age-matched non-PD controls (healthy donors (HD)) by flow cytometry using the antibody specific to a conservative epitope shared by constitutive Hsc70 and stress-induced Hsp70-1 proteins (Supplementary Figure S1a). No significant difference in the HSP70basal (Hsc70 + Hsp70-1) levels between PD patients and HDs was observed in our cohorts (Figure 1a). Additionally, we analyzed a reversible elevation of the HSP70 level in response to mild heating (HSP70heat) in PBMC. In our previous study, we showed that this increased HSP70 level was not associated with de novo protein synthesis and presumably reflected an increase in the accessibility of the epitope for the antibody [35]. In this work, we also evaluated the difference between HSP70heat and HSP70basal values (ΔHSP70 = HSP70heat − HSP70basal). HSP70heat levels, as well as ΔHSP70 values were comparable in PBMC of PD patients and HDs (Figure 1a). Significant positive correlations were revealed between HSP70basal level and HSP70heat level in PBMC in both patients with PD and HDs (Figure 1b,c). Thus, we did not observe changes in the level of intracellular HSP70 chaperones in PBMC from mild to moderate PD patients.

### 3.2. Transcriptional Activity of Stress-Associated HSPA Genes Is Increased in PBMC of PD Patients

Generally, the intracellular protein level is dependent on the accumulation and stability of mRNA. However, in some cases, mRNA accumulation may weakly correlate with protein amount because of a discrepancy between transcriptional and translational activity [36]. Taking this into consideration, we examined the basal transcriptional activity of the *HSPA* genes encoding HSP70 proteins in PBMC from PD patients and HDs by real-time PCR.

**Figure 2:**
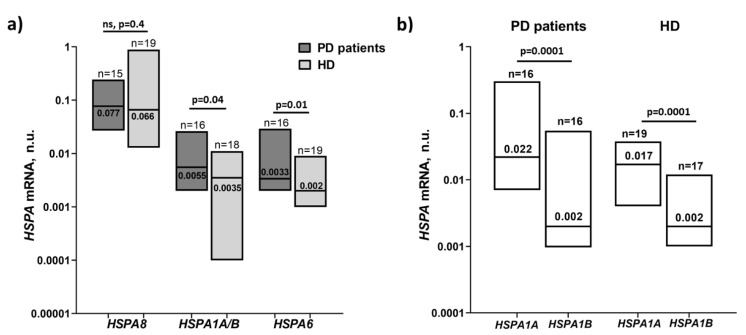
Basal transcriptional activity of *HSPA8*, *HSPA1A/B*, *HSPA1A*, *HSPA1B*, and *HSPA6* genes in PBMC, analyzed by real-time quantitative reverse transcription PCR (qRT-PCR). (**a**) Comparison of the *HSPA8*, *HSPA1A/B,* and *HSPA6* mRNA levels between PD patients and HDs. (**b**) The mRNA levels of the *HSPA1A* and *HSPA1B* genes in PBMC of PD patients and HDs. Data of mRNA levels were normalized to that of β-actin; n.u., normalized units; data are presented as floating bars (min to max) with the median; solid lines, the median.

The level of transcriptional activity of the *HSPA8* encoding Hsc70, which is constitutively expressed in all cells, did not differ between PD patients and HDs in our cohorts (Table 2, Figure 2). These results correspond to the data of the protein level analysis (Figure 1a). We also measured the basal transcriptional activity of *HSPA1A/B* and *HSPA6* encoding stress-induced proteins Hsp70-1 and Hsp70B′, respectively. We observed a significant increase in the basal transcriptional activity of stress-induced *HSPA1A/B* and *HSPA6* genes in PBMC from PD patients compared to HDs (Table 2, Figure 2a). To discriminate *HSPA1A* and *HSPA1B* genes, which encode the protein isoforms Hsp70-1a and Hsp70-1b, respectively, we designed primers to the 3′-UTRs (3′-untranslated regions) of the genes, as there is sequence variation observed between the genes in this region (Supplementary Figure S2).

Expression analysis of separate genes *HSPA1A* and *HSPA1B* in PBMC revealed that the increased *HSPA1A/B* expression observed earlier in PD patients was possible due to the *HSPA1A* isoform contribution, while the medians of the *HSPA1B* expression levels were similar in the comparison groups (Table 2, Figure 2a). However, the observed difference in the *HSPA1A* transcriptional activity was insignificant between PD patients and HDs (Figure 2b). Positive correlations were found between the *HSPA1A/B* and *HSPA6* gene expression and also between the *HSPA1A* and *HSPA6* gene expression levels in PBMC from both PD patients (r = 0.61, *p* = 0.013; r = 0.59, *p* = 0.0078, respectively) and HDs (r = 0.55, *p* = 0.017; r = 0.69, *p* = 0.002, respectively) (Supplementary Figure S3).

To analyze the obtained results in the context of their specific relationship to PBMC, we examined the transcriptional activity of the *HSPA* genes in polymorphonuclear leukocytes (PMN), which are widespread cells in the bloodstream. No differences were found in the expression levels of *HSPA8, HSPA1A/B*, *HSPA1A, HSPA1B,* and *HSPA6* genes in PMN in PD patients and HD groups (Table 2). Thus, the observed increase in transcriptional activity of the *HSPA1A/B* and *HSPA6* genes is specific for PBMC but not for PMN during PD.

### 3.3. An Increased Level of Spontaneous Apoptosis Is Associated with an Increased Accumulation of p62 Protein in PBMC from PD Patients

**Figure 3:**
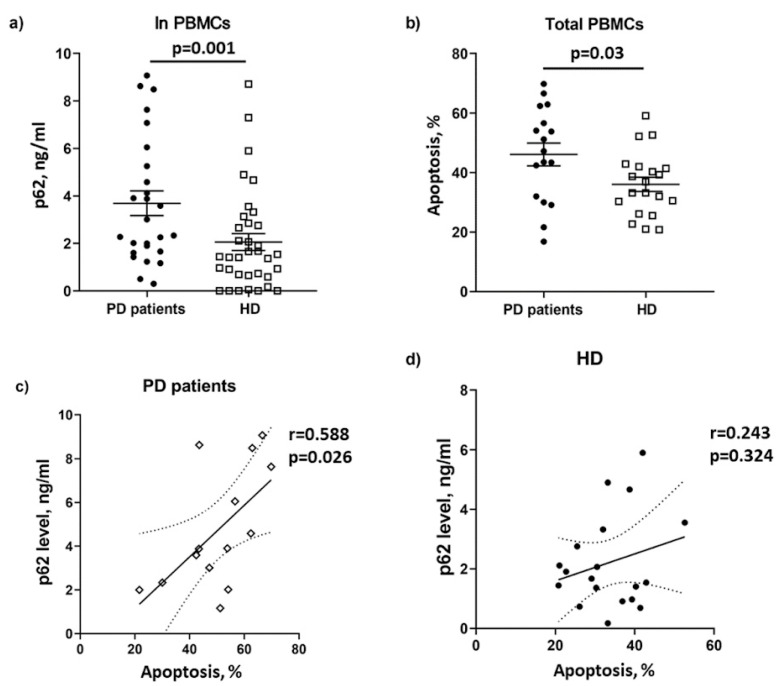
The intracellular level of p62 protein and the percentage of spontaneous apoptosis in PBMC of PD patients and HDs and their correlation. (**a**) Differences in the intracellular level of p62 protein (ng/mL) between PD patients and the HD group. (**b**) The percentages of cells undergoing spontaneous apoptosis among PBMC from PD patients and HD. (**c**) Correlation analysis between apoptotic cell percentage and p62 level (ng/mL) in PBMC from PD patients (**d**) from HDs. Solid lines, mean ± SEM. Pearson’s correlation coefficient was used; dotted line, 95% confidence interval.

We measured the basal level of p62 protein to analyze the macroautophagic flux [21] in PBMC isolated from PD patients and compared it with the HD cohort. The content of intracellular p62 protein was significantly higher in PD patients compared to HDs, which can indicate the regress of autophagic flux in PBMC in PD (Figure 3a). Since autophagy is crucial in cell death decisions [37], we analyzed spontaneous apoptosis of PBMCs in the examined groups. The percentage of apoptotic cells among PBMC was increased in PD patients compared to HDs (Figure 3b). A moderate, positive correlation between the basal intracellular content of p62 and the level of spontaneous apoptosis was found in PD patients, whereas such a relationship was absent in the group of HDs (Figure 3c,d). We hypothesized that the enhanced accumulation of p62 protein in PBMC from PD patients is a prerequisite for their facilitated apoptosis.

### 3.4. Analysis of Diagnostic Efficiency of the Stress-Associated HSPA Genes’ Expression and the p62 Protein Level as Differential Biomarkers of PD

**Figure 4:**
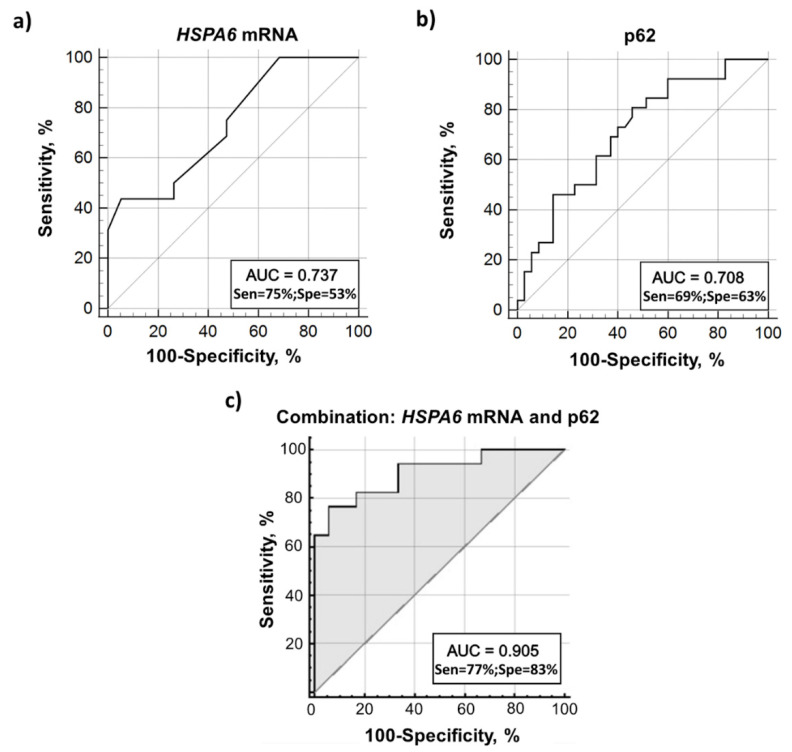
ROC curve analysis showing *HSPA6* gene expression levels and p62 protein levels as potential biomarkers of PD. (**a**) The *HSPA6* mRNA levels in PBMC showed 75.0% sensitivity and 52.63% specificity in distinguishing PD. (**b**) The p62 protein level in PBMC showed 69.23% sensitivity and 62.86% specificity in distinguishing PD. (**c**) Combination of *HSPA6* mRNA and p62 protein showed 77% sensitivity and 83% specificity in distinguishing PD. ROC, receiver operating characteristic; AUC, the area under the ROC curve; Sen, sensitivity; Spe, specificity.

To evaluate the utility of *HSPA1A/B* and *HSPA6* mRNA levels and p62 protein levels in PBMC in discriminating individuals with PD from HDs, we performed receiver operating characteristic (ROC) curve analysis. The *HSPA6* mRNA level with a cutoff value of 0.003 n.u. and p62 protein level with a cutoff value of 1.9 ng/mL displayed both moderate sensitivity and relatively low specificity (Figure 4a,b). The biomarker based on the *HSPA1A/B* mRNA level could not significantly distinguish PD patients from HDs (Supplementary Figure S4).

Next, we applied a classifier based on the K Nearest Neighbors’ (KNN) method using both p62 protein and *HSPA6* mRNA levels for distinguishing between PD patients and age-matched HDs. The analysis demonstrated intermediate sensitivity and high specificity of this combination (Figure 4c). Thus, the combination of p62 protein and *HSPA6* mRNA levels may help to distinguish PD patients from non-PD individuals and can serve as a potential PD biomarker.

## 4. Discussion

PD-related degenerative disorder is associated by the disruption in the proteostasis network in brain cells including intracellular pathways that control the chaperone-assisted protein folding, trafficking, degradation, and autophagy–lysosome-associated processes. CMA and macroautophagy (autophagy) are based on lysosomal degradation of intracellular waste/damaged proteins and organelles; different key regulators and signaling pathways are described for these processes. In general, in response to different stimuli, several mechanisms of post-translational and transcriptional regulation of autophagy are initiated. In this study, we focused on two components of the proteostasis system in extraneuronal cells in PD: the HSP70 family chaperones, including their stress-associated members, and macroautophagy mediator p62.

In our cohort of patients with idiopathic PD, we did not reveal any significant reduction in the HSP70 pool (Hsc70 + Hsp70-1) basal level analyzed by flow cytometry in freshly isolated PBMC, in comparison to HDs. It was considered that the Hsc70 protein was prevalent in intact PBMC compared to other members of the HSP70 family [14,34]. Indeed, the evaluation of the basal Hsp70-1 level separately revealed only trace amounts of the stress-induced Hsp70-1 compared to Hsc70 protein in intact and mildly heated PBMC from HDs (Supplementary Figure S1b).

Intracellular HSP70 proteins practically do not exist in vivo in the substrate-free state. They can be found in various complexes interacting with other proteins, nucleotides, and membranes, and they form oligomers [38,39]. In non-denatured conditions, HSP70 protein in a bound form can be only partially detected by flow cytometry because some epitopes would be hidden from the specific antibodies. In our earlier work, we described a reversible elevation of the HSP70 level, which can be detected after mild heating of the cells (HSP70heat). The HSP70heat level was not associated with de novo protein synthesis and was apparently attributed to a better accessibility of certain epitopes of the HSP70 substrate-binding domain to the antibody [35]. Thus, HSP70heat and ΔHSP70 (the difference between HSP70heat and HSP70basal) values correspond predominantly to the amount of uncoupled forms of the HSP70 proteins. The intracellular HSP70 level has an age-dependent pattern: it was shown to be increased with aging in human blood mononuclear cells [40]. From this point of view, malformed proteins accumulated with age require more HSP70 assistance and, consequently, the delta value (ΔHSP70) can be significantly increased with age, at least in neutrophils [35]. Despite the fact that PD is associated with aging and defects in protein folding ΔHSP70, values did not differ between PD patients and HDs either in the population of PMN (Supplementary Figure S5) or in PBMC in our cohorts. In contrast to the HSP70heat level, the ΔHSP70 value did not correlate with the HSP70basal level in either PD patients or age-matched HD (Figure 1b,c).

It was shown earlier that Hsc70 protein and Hsc70-coding mRNA levels are significantly reduced in the PBMC of PD patients [41,42], which is inconsistent with our data. The discrepancies in the results might be explained by different methods used for HSP70 detection. Our analysis did not reveal any change in the transcriptional activity of the *HSPA8* gene, encoding Hsc70, in PBMC from the PD patients compared to the healthy individuals, which corresponds to and corroborates our results for HSP70 protein levels. Alternatively, the discrepancy between the studies may be explained by the different clinical characteristics of idiopathic PD cases included in the investigations. Since idiopathic PD is now considered to have a complex etiology involving multiple factors, such as lifestyle, genetics, and environment [43], the cohorts of donors with PD vary significantly in different studies. Possibly the detectable decrease in the level of intracellular Hsc70 protein in PBMC, which did not exceed 20% [41], suggests that the demographic and clinical characteristics of the patients’ cohort can be a critical factor for detecting deficiency in Hsc70, a regulator of chaperone-assisted protein folding in these cells. This point of view was confirmed by the absence of any correlation between Hsc70 expression level in PBMC and the progression level of PD (UPDRS III score and disease duration) [41] and, in summary, implied that the level of Hsc70 in PBMC cannot be used as a biomarker for PD.

In contrast to *HSPA8*, we showed that the expression of the stress-induced *HSPA* genes was increased degrees in PBMC from PD patients (Figure 2a). *HSPA1A/B* gene transcriptional activity was significantly higher in PD patients compared to healthy age-matched individuals. Possibly this increase was contributed mainly by the *HSPA1A* isoform (Figure 2b), which, according to the literature data [14] and to our results, is the most transcriptionally active among the *HSPA1* genes. Of note, *HSPA1B* gene expression levels varied to a great extent in the cohort of PD patients (Figure 2b). Thus, in individuals with a considerably high expression level of this, the *HSPA1B* isoform could also contribute to the increased *HSPA1A/B* expression level in the PD group. Earlier, an SNP-dependent variability of *HSPA1B* transcriptional activity was demonstrated [44]. In our study performed on a cohort from the Russian population, higher *HSPA1B* expression levels were found in PBMC from donors homozygous in certain genes compared to heterozygotes. The *HSPA6* transcript level was also significantly increased in PD patients compared to HDs. Initially, the main stress-induced *HSPA6* and *HSPA1A* genes were considered to be derived from the same ancestral gene [45].

The increased *HSPA6* gene expression is known to be an indicator of strong cell stress, often exhibiting distinct features, in targeting protein substrates that are not observed for *HSPA1A* [46]. The simultaneous and possibly interrelated increase in the mRNA level of *HSPA1A/B* genes, specifically *HSPA1A* and *HSPA6,* represented in circulating mononuclear cells was demonstrated in our study via strong positive correlations between these values in PD patients and the HD group (Supplementary Figure S3). We revealed that, of these shifts that are specific for PBMC, transcriptional activity of *HSPA1A/B* and *HSPA6* was not increased in PMN in PD cases, even considering the fact that in non-stressed conditions PMNs were characterized by higher *HSPA1A/B* and *HSPA6* activity than PBMC (Supplementary Figure S6) [34]. We concluded that the stress-induced HSPA response in PBMC could be a consequence to the cellular stress observed in PD. The involvement of inflammatory-mediated stress (oxidative stress) in PD is confirmed by numerous studies [47,48,49]. The consequences of oxidative stress on lysosomal degradation in PBMCs from PD individuals were described [24].

Macroautophagy is a constitutively active process in most cells of the human body and is referred to as “basal autophagy.” We revealed that the level of macroautophagy-related cytosolic component, intracellular p62 protein, was significantly higher in PD patients compared to HDs. Up until now, there has been no data describing the p62 level as a peripheral marker of PD. The p62 protein was detected in Lewy bodies from post-mortem brain tissue of PD patients, further supporting the involvement of macroautophagy in the progression of PD [50]. Recently, the overexpression of the *P62/SQSTM1* gene in PD patients’ PBMC was demonstrated [51]. In our work, the p62 protein level in PBMC positively correlated with the spontaneous apoptosis level in the PD patient group. Several mechanisms describe how autophagy–lysosome pathway plays a critical role in apoptosis, for example, via p62-mediated regulation of polyubiquitination and aggregation for C-terminal cytosolic Fas/CD95 [52] or via activation of caspase-8 [53]. In relation to PD, peripheral blood CD4(+) T cells have been shown to have increased susceptibility to Fas-induced apoptosis [54]. Since p62 takes part in both autophagy and apoptosis pathways, the increased level of p62 in PD patients may enhance the predisposition of PBMC to spontaneous apoptosis.

The involvement of p62 in macroautophagy and in the progression of PD may be considered in various aspects. The signaling adapter p62 is a mediator of important cellular functions through its ability to interact with various signaling messengers, and it is an integral part of the mTORC1 complex, which is necessary for autophagy signaling activation [55]. The interaction of the lipidated form of the LC3 protein (LC3-II) and p62 is a central event of autophagy and is crucial for the autophagosome forming [56]. LC3-I to LC3-II conversion is elevated in PBMCs [24], and LC3 gene expression as well as LC3-II protein levels are significantly increased in the leukocytes of PD patients, indicating autophagosome accumulation [6]. On the other hand, the number of autophagy vacuoles per cell in PBMC of PD patients is decreased compared with healthy subjects [57]. The modulation of intracellular signaling implicated in neuronal cell survival, growth, proliferation, and metabolism in PD indicates that p62-involved autophagy activity is regulated at post-translational and transcriptional levels [58]. For example, expression of the substrate-capturing p62/SQSTM1 gene is strongly dependent on the transcription factor EB [59]. Moreover, TFEB-mediated pathways are involved in the resistance to oxidative stress-promoting lysosomal biogenesis and regulating autophagic proteins [60]. P62-related function is not only limited to turnover of autophagic lysosomes. The inability to eliminate p62 through autophagy can lead to a toxic increase in oxidative stress and DNA damage in some tissues [61,62]. Several biologically active compounds are capable to modulate the signaling pathways mentioned above and possess anti-parkinsonian activity, mainly through anti-inflammatory and anti-oxidant properties [63,64,65,66]. Considering that the stress-inducible cellular protein p62 serves as a selective autophagy receptor for recruitment to the lysosome and implicates in the oxidative stress response with regard to mTORC1 activation, we can hypothesize that the increased accumulation of p62 detected by us in PBMC of PD patients may happen due to disturbance of the upstream p62-regulating pathways.

Based on ROC analysis, we concluded that *HSPA1A/B* gene expression cannot significantly distinguish PD from HD and an elevated level of *HSPA6* mRNA or intracellular p62 protein level alone may not be considered as reliable PD biomarkers because of insufficient specificity (Supplementary Figure S4, Figure 4a,b). At the same time, the basal transcriptional activity of *HSPA6* in combination with the p62 protein level has potential diagnostic value in PD discrimination (Figure 4c). Obviously, proteostasis-linked intracellular HSP70/Hsc70 levels in PBMC are not reliable and specific markers of PD compared to their levels in the substantia nigra lesions in PD. On the other hand, elevated *HSPA1A/B* and *HSPA6* gene expression in PBMC can indicate the cell stress response on the background of PD. Thus, we assume that the combined measuring of *HSPA6* gene expression and p62 protein level as a complex biomarker may have diagnostic significance for distinguishing patients with PD.

## Figures and Tables

**Table 1:** PD patients and HD cohort characteristics. N is the number of donors. SD, standard deviation; n/a = not applicable.

	PD Patients *n* = 26	HD *n* = 36
Age (Median)	57.5	54.6
Male (*n*)	8	12
MDS-UPDRS motor score (mean ± SD)	24.9 ± 8.4	n/a
Hoehn and Yahr (mean ± SD)	2.8 ± 0.5	n/a
Duration of PD, years (mean ± SD)	7 ± 0.8	n/a

**Table 2:** Normalized mRNA levels of *HSPA* genes (medians) in PBMC and PMN in PD patients compared to HDs.

**The *HSPA* Genes in PBMC**	**PD Patients *n* = 15**	**HD *n* = 19**	***p*-Value**
*HSPA8*	0.066	0.077	0.4
*HSPA1A/B*	0.0055	0.0035	0.04
*HSPA1A*	0.021	0.017	0.1
*HSPA1B*	0.002	0.002	0.9
*HSPA6*	0.0033	0.002	0.01
**The *HSPA* Genes in PMN**	**PD Patients *n* = 13**	**HD *n* = 16**	***p*-Value**
*HSPA8*	0.014	0.015	0.8
*HSPA1A/B*	0.014	0.015	0.6
*HSPA1A*	0.058	0.055	0.4
*HSPA1B*	0.002	0.003	0.8
*HSPA6*	0.018	0.021	0.8

## Supplementary Materials

The following supporting information can be downloaded at https://www.mdpi.com/article/10.3390/biom12040493/s1. Figure S1: HSP70 levels in PBMC were measured by flow cytometry. Representative staining of three independent experiments is shown; Figure S2: The design of primers for discrimination of the HSPA1A and HSPA1B expression; Figure S3: A correlation analysis between stress-induced HSPA gene expression in PBMC in PD patients and HDs; Figure S4: ROC curve analysis of *HSPA1A/B* gene expression levels as potential biomarkers of PD; Figure S5: ΔHSP70 values calculated by subtraction of HSP70basal from HSP70heat levels in PMN in 14 PD patients and 14 HDs; Figure S6: Comparison of the stress-induced *HSPA1A*, *HSPA1B* and *HSPA6* gene expression between two cell subsets: PBMC and PMN. Table S1: Oligonucleotide primers used in the study.
