# Supplementary material for: Alterations in Proteostasis System Components in Peripheral Blood Mononuclear Cells in Parkinson Disease: Focusing on the HSP70 and p62 Levels
Source: Biomolecules. 2022 Mar 24;12(4):493. doi: 10.3390/biom12040493 (PMC9030208; doi:10.3390/biom12040493)
Supplement: Supplementary file 1 [file biomolecules-12-00493-s001.zip › biomolecules-1626691-supplementary/Supplementary File.pdf]

Supplementary File

Materials and Methods

Supplementary Table S1. Oligonucleotide primers used in the study.

Gene	Forward primer	Reverse primer	Amplicon length
HSPA8	TGCTGCTCTTGGATGTCACT	AAGGTCTGTGTCTGCTTGGT	112bp
HSPA1A/B	AGGTGCAGGTGAGCTACAAG	CTCGGCGATCTCCTTCATC	92bp
HSPA1A	TTTTTCCGGTTTCTACATGCAG	CAACTTAAAAAATGGCCTGAGT	96bp
HSPA1B	TCTTTAGTATGTTTGTCTTTGAGGTGG	TGGCAGTGTTGATTCATTTAAAGG	239bp
HSPA6	ACCCAGGTGTATGAGGGTGA	TCTATCTGGGGGACTCCACG	107bp
β-actin	CACCACACCTTCTACAATGAG	GTCTCAAACATGATCTGGGTC	119bp

Results

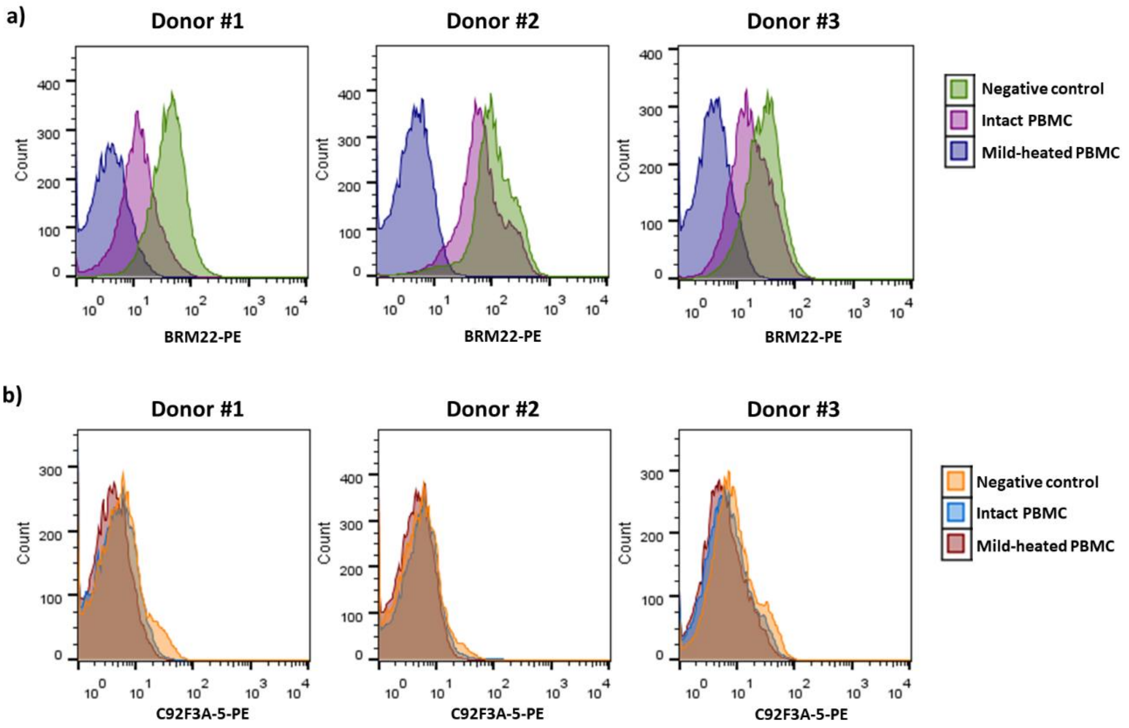

Supplementary Figure S1. HSP70 levels in PBMC were measured by flow cytometry. Representative staining of three independent experiments is shown. a) The experiments were carried out using BRM22 monoclonal antibody, recognizing both stress-induced Hsp70 and constitutive Hsc70. b) The experiments were carried out using specific antibodies C92F3A-5, recognizing stress-induced Hsp70.

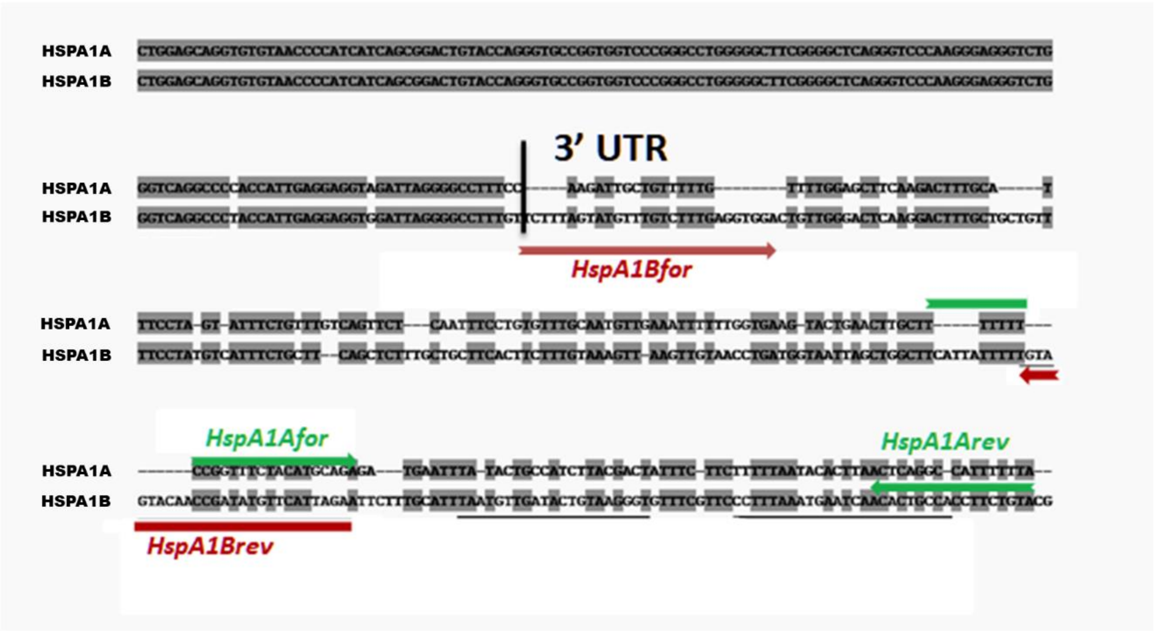

Supplementary Figure S2. The design of primers for discrimination of the *HSPA1A* and *HSPA1B* expression. The alignment of *HSPA1A* mRNA (RefSeq NM_005345) and

HSPA1B mRNA (RefSeq NM_005346) is given. Complementary nucleotides are designated in gray. 3’ UTR - 3’ untranslated region.

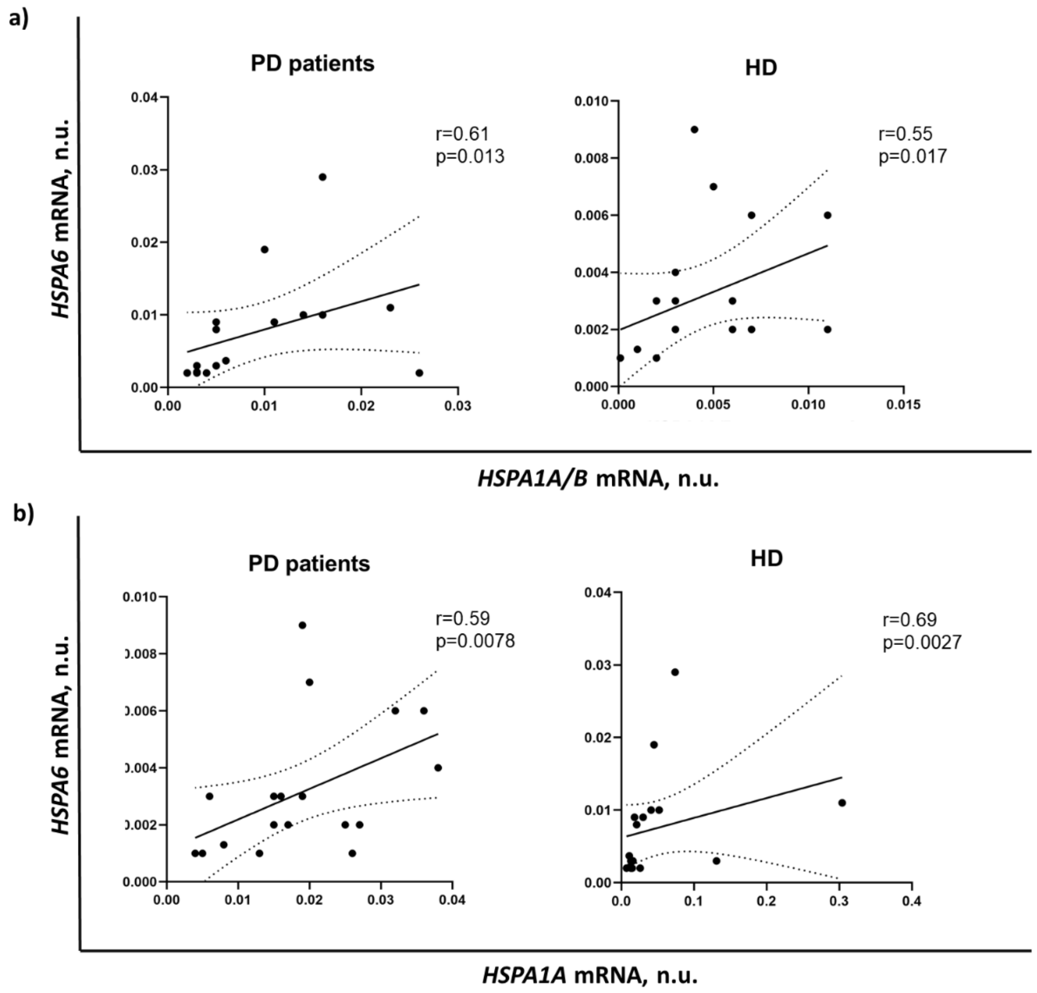

Supplementary Figure S3. A correlation analysis between stress-induced *HSPA* gene expression in PBMC in PD patients and HDs (**a**) *HSPA1A/B* vs *HSPA6* mRNA levels; (**b**) *HSPA1A* vs *HSPA6* mRNA levels. Data of mRNA levels were normalized to that of β-actin; n.u.- normalized units.

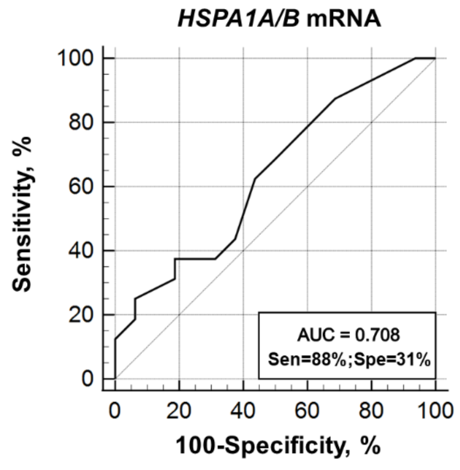

Supplementary Figure S4. ROC curve analysis of *HSPA1A/B* gene expression levels as potential biomarkers of PD. The *HSPA1A/B* mRNA in PBMC showed 88.0% sensitivity and 31% specificity.

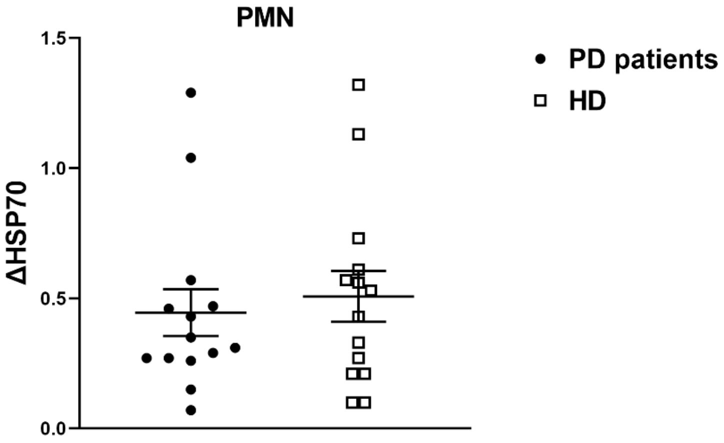

Supplementary Figure S5. ΔHSP70 values calculated by subtraction of HSP70basal from HSP70heat levels in PMN in 14 PD patients and 14 HDs.

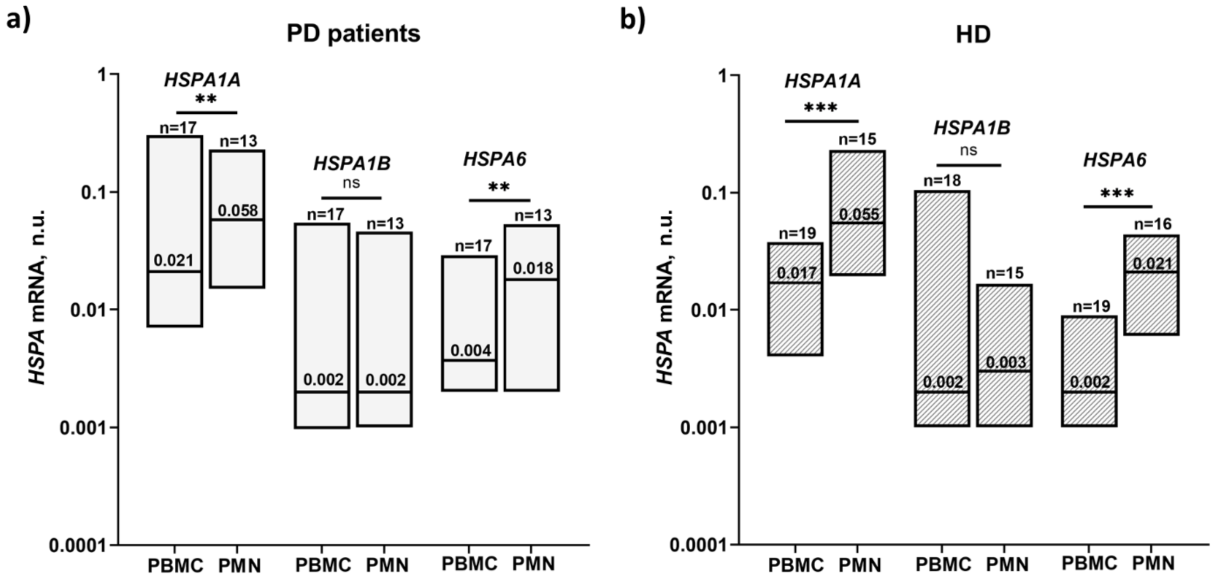

Supplementary Figure S6. Comparison of the stress-induced HSPA1A, HSPA1B and HSPA6 gene expression between two cell subsets: PBMC and PMN (**a**) isolated from PD patients (**b**) isolated from HDs. Data is presented as floating bars (min to max) with the median. ** $p < 0.01$, *** $p < 0.001$.
